# Synthesis of Reduced Grapheme Oxide as A Platform for loading β-NaYF_4_:Ho^3+^@TiO_2_Based on An Advanced Visible Light-Driven Photocatalyst

**DOI:** 10.1038/s41598-017-14018-3

**Published:** 2017-10-23

**Authors:** Zihong Fan, Tianhui Wu, Xuan Xu

**Affiliations:** 1 Chongqing Key Laboratory of Catalysis and Functional Organic Molecules, College of Environmental and Resources, Chongqing Technology and Business University, Chongqing, 400067 China; 2 Key Laboratory of Three Gorges Reservoir Region’s Eco-Environment, Ministry of Education, Chongqing University, Chongqing, 400045 China

## Abstract

In this paper a novel visible light-driven ternary compound photocatalyst (β-NaYF_4_:Ho^3+^@TiO_2_-rGO) was synthesized using a three-step approach. This photocatalyst was characterized using X-ray diffraction, Raman scattering spectroscopy, scanning electron microscopy, energy-dispersive X-ray spectroscopy, Transmission electron microscopy, X-ray photoelectron spectroscopy, fluorescence spectrometries, ultraviolet-visible diffuse reflectance spectroscopy, Brunauer–Emmett–Teller surface area measurement, electron spin resonance, three-dimensional fluorescence spectroscopy, and photoelectrochemical properties. Such proposed photocatalyst can absorb 450 nm visible light while emit 290 nm ultraviolet light, so as to realize the visible light-driven photocatalysis of TiO_2_. In addition, as this tenary compound photocatalyst enjoys effecitve capacity of charge separation, superior durability, and sound adsorb ability of RhB, it can lead to the red shift of wavelength of absorbed light. This novel tenary photocatalyst can reach decomposition rate of RhB as high as 92% after 10 h of irradiation by visible-light Xe lamp. Compared with the blank experiment, the efficiency was significantly improved. Recycle experiments showed that theβ-NaYF_4_:Ho^3+^@TiO_2_-rGOcomposites still presented significant photocatalytic activity after four successive cycles. Finally, we investigated visible-light-responsive photocatalytic mechanism of the β-NaYF_4_:Ho^3+^@TiO_2_-rGO composites. It is of great significance to design an effective solar light-driven photocatalysis in promoting environmental protection.

## Introduction

Semiconductor photocatalysis has attracted worldwide attention due to its potential inresolving the energy and environmental-related problems through increasing solar energy conversion^1,2^. Titanium dioxide (TiO_2_), as one of the most promising photocatalysts, has been widely adopted for degrading inorganic and organic pollutants because of its strong oxidizing capacity under ultraviolet (UV) light, extraordinary chemical stability, sound biocompatibilty and environmental friendliness^3–6^. However, due to the wide bandgap of TiO_2_ (Eg = 3.0–3.2 eV), only the UV light with wavelength less than 387 nm, which accounts for ca. 5% of total solar energy, could produce effective photocatalytic activity^7^. While the visible light (Vis) and near-infrared (NIR), which account for ca. 48% and ca. 45%, respectively, are not valid for photocatalysis^8^. Furthermore, the fast recombination of charge carriers will significantly reduce the catalytic activity in practical applications^9,10^. As a result, further innovative researches are needed to realize large scale practical application of TiO_2_-based photocatalysts for solving environmental and energy problems.

To resolve this problem, many attempts have been made to improve the photocatalytic activity of TiO_2_ by inhibiting the recombination of photogenerated electron–hole pairs and extending the absorption range of TiO_2_ towards Vis region, so as to realize a better use of solar energy^11–13^. For example, to extend the bandgap of TiO_2_ towards Vis, conventional methods such as introduction of metals^14^ or nonmetals^15^, surface modification^16^, and structure optimization^17^ have been used to extend the bandgap of TiO_2_ toward Vis energies. In these processes, noble metal doping, composite semiconductor, and holes scavenger can effectively inhibit the recombination of electrons and holes. Although these methods can extend the absorption of TiO_2_ to the Vis region and inhibit the recombination of photogenerated electron–hole pairs, many new defects may occur when adjusting the internal lattice and band structure to shorten the bandgap of TiO_2_ and let the electrons pass quickly^18^. For instance, with the implementation of these methods, it will cause decreased stability and service life of TiO_2_, as well as low activity under UV light^19,20^. Thus, other methods must be sought to extend the absorption range of TiO_2_ and thus to enhance the photocatalysis activity.

Recently, lanthanide-doped upconversion (UC) nanophosphors have attracted tremendous interest because of their narrow emission bands, long luminescence lifetime, as well as their physicochemical properties that are conductive to extending the absorption range of TiO_2_ edge up to the Vis region or even to the NIR region by UC luminescence of rare earths^21–24^. In order to efficiently utilize the valuable UC energy transferred from the excited UC core, a coupling model was proposed to increase the luminous efficiency, of which the core-shell structure has potential in protecting the UC core from surface quenching and in increasing the efficiency of transferring energy to the photocatalyst shell^25–28^. For examples, the preparation of (α, β)-NaYF_4_:Yb^3+^,Tm^3+^@TiO_2_, YF_3_:Yb^3+^,Tm^3+^@TiO_2_, and Y_2_O_3_:Yb^3+^ has been reported, wherein the core-shell composite of UC@TiO_2_can emit UV and Vis and show an effective photocatalysis under NIR light^29–33^. However, Yb^3+^, an important sensitizer, has only one excitation at 980 nm in the NIR region. Its UC efficiency is limited by NIR radiation source excitation, which is mainly due to its extremely weak absorption in the NIR wavelength range^34^. We conducted researches on core-shell microcrystal of β-NaYF_4_:Ho^3+^@TiO_2_ and on the use of Vis as a photocatalyst^35^. This combination takes advantages of Vis and NIR which accounts for ca. 48% and 45% of total solar energy, respectively. However, there still remains some problems about core–shell structure of UC@TiO_2_.For example, the efficient utilization of light is limited, because the light cannot penetrate the cores of UC nanocrystals. Moreover, the fast recombination of photogenerated electron–hole pairs of UC@TiO_2_should not be ignored as they can affect the photocatalytic efficiency^36^. Therefore, it is needed to seek other methods to solve these problems.

Graphene (GR) and reduced graphene oxide (rGO) have emerged as attractive candidates for constructing GR–based materials because of their several valuable characteristics^37–40^. First, GR materials exhibit high electron mobility (200, 000 cm^2^/V) and extended π-electron conjugation, therefore GR is a good material for transporting electrons and stabilizing extraneous electrons. In addition, GR enjoys high specific surface area (2630 m^2^/g) and unique flexible sheet-like structure. Recently, great attention has been attracted by TiO_2_–GR, which displays the capacity of significantly improving photoelectrochemical catalytic activity^41,42^. As of now, there have been lots of reports on TiO_2_–GR composites. Researches indicate that GR can not only increase the adsorption surface of the catalyst because of its high theoretical specific surface area, but also can improve the adsorption performance of TiO_2_ by chemical adsorption^42^. The TiO_2_@GR composite has high light transmittance and zero-bandgap of GR. Moreover due to the excellent electrical conductivity for electrons storing and shuttling (flexible sheet-like structure), it can inhibit the recombination of photogenerated electron–hole pairs^43–48^. Moreover, the formation of Ti–O–C bonds can expand the light absorption to longer wavelengths^49^. After absorbing photons on the surface of GR, the electrons are injected into the TiO_2_ conduction band to form a reactive exciton (·OH, O_2_^−^) for degradation of organic pollutants. Despite all the advantages, these TiO_2_@GR composites are subjected to a low-usage of natural sunlight, which will limit the photocatalytic activity under solar irradiation^50^.

In general, there are at least two major challenges which may lead to the difficulty in realizing the large scale practical application of TiO_2_ photocatalysis. One is how to improve the utilization rate of solar light, which can be realized through extending the absorption range of TiO_2_ using UC nanophosphors^36,51,52^. The other one is how to inhibit the recombination of photogenerated electron–hole pairs. The GR/rGO is expected to serve as an electron collector and transporter, which can accept and shuttle the electrons generated from the semiconductors, and thus preventing recombination and prolonging lifetime of the photogenerated electron–hole pairs. A series of studies have been carried out in these two aspects. Long and Wang^36,52^ studied on the preparation of YF_3_:Yb^3+^,Tm^3+^/P25/GR and α-NaYF_4_:Yb^3+^,Tm^3+^/TiO_2_/rGO composites that emit UV and Vis light under 980 nm excitation and show a higher efficiency as compared with a physical mixture. Nevertheless, following questions are still existed. Firstly, the individual components (i.e. UC materials, P25–TiO_2_ and GR) are only physically mixed without well-defined nanostructures. Secondly, the nanosized YF_3_ and α-NaYF_4_ have relatively low UC abilities compared with the microsized hexagonal phase (β-NaYF_4_)^53^. So the technology can still be improved. Wang *et al*.^51^ studied on the preparation of β-NaYF_4_:Yb^3+^, Tm^3+^/N-P25/GR composites as an advanced NIR and Vis-driven UC photocatalyst. However, the individual components are only physically mixed without well-defined nanostructures. As a result, the photocatalytic activity under NIR is relatively low and unsatisfactory. Meanwhile, the existing researches are mainly focused on the sensitization agent. Yb^3+^, an important sensitizer, has only one excited electronic state around the near-infrared region (980 nm). Therefore, the excitation sources for Yb^3+^-doped UC materials are in the near-infrared range, rather than in the Vis range. It is difficult to convert near-infrared radiation to higher energy UV radiation, because the UC process requires the absorption of three photons or more. As a result of this requirement, UC efficiency of Yb^3+^-doped UC materials is low when a near-infrared radiation source is used. However Ho^3+^ single-doped UC materials have received little attention, and studies have indicated that Ho^3+^ single-doped UC nanoparticles can absorb Vis light and emit UV radiation. This combination also takes advantage of the Vis light which accounts for ca. 48% of total solar energy.

In this paper, we integrated the above-mentioned strategies and demonstrated the rGO–assisted core–shell structure β-NaYF_4_:Ho^3+^@TiO_2_ could act as a new Vis-driven photocatalyst for the first time. Benefiting from the high specific surface area and the flexible sheet-like structure, rGO emerged as an excellent platform on which the core–shell microcrystal β-NaYF_4_:Ho^3+^@TiO_2_ can be load to form the ternary composite β-NaYF_4_:Ho^3+^@TiO_2_–rGO. In this photocatalyst, it is expected to emit UV light after absorbing Vis light of the solar spectrum during the loading process of UC microcrystals, meanwhile the optical response of the β-NaYF_4_:Ho^3+^@TiO_2_–rGO will be enhanced from UV to Vis. In addition to the microcrystals catalyst β-NaYF_4_:Ho^3+^@TiO_2_–rGO which can improve the utilization of sunlight, rGO can inhibit the recombination of photogenerated electron–hole pairs and enhance the adsorption capacity of the photocatalyst. In addition, we conducted detailed discussion on the Vis responsive photocatalytic mechanism of β-NaYF_4_:Ho^3+^@TiO_2_–rGO.

## Results and Discussion

### Structure and morphology characterizations

**Figure 1:**
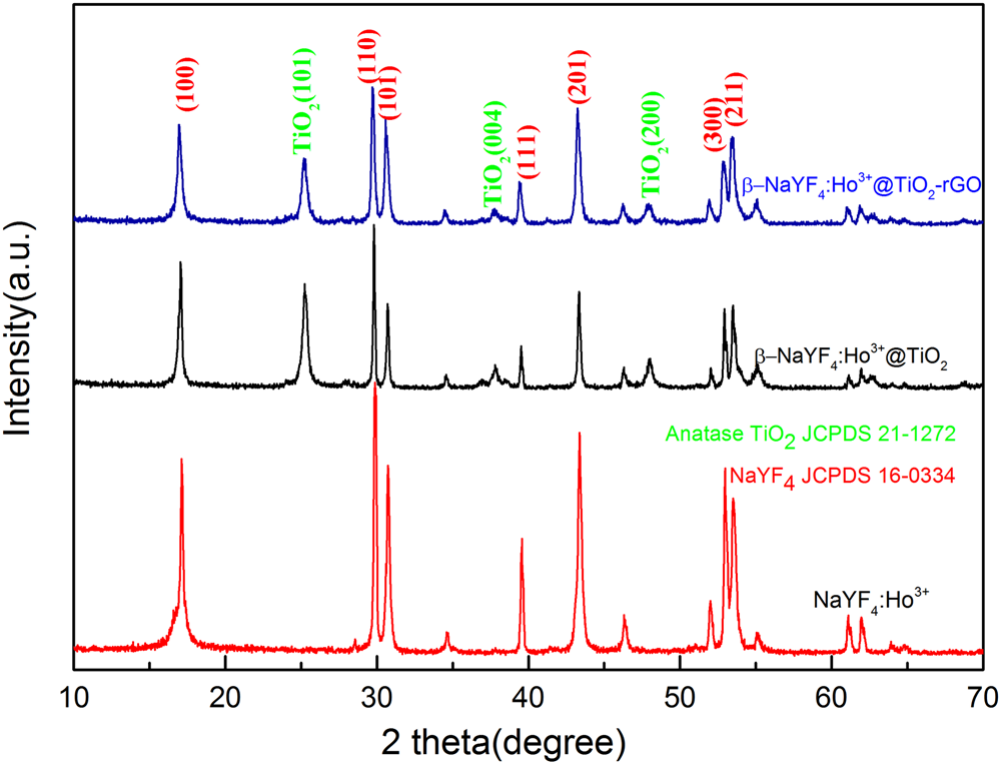
The XRD patterns of β-NaYF_4_:Ho^3+^, β-NaYF_4_:Ho^3+^@TiO_2_, and β-NaYF_4_:Ho^3+^@TiO_2_-rGO.

Figure 1 shows the XRD patterns of β-NaYF_4_:Ho^3+^, β-NaYF_4_:Ho^3+^@TiO_2_ and β-NaYF_4_:Ho^3+^@TiO_2_-rGO, respectively. From the XRD images of the three samples in Figure. 1, we can see that diffraction peaks are very sharp. This indicates that the samples resulted from hydrothermal synthesis are of high purity and excellent crystallinity, and such excellent crystallinity plays a vital role in determmining the photocatalysis efficiencies^54^. In addition, we can see that the diffraction peaks of UC material at 17.2°, 30.1°, 30.8°, 43.5°, and 53.7° can perfectly match with the standard card of β-NaYF_4_(JCPDS no. 16–0334), without the occurrence of impurity-contained diffraction peak. This indicates that the high purity UC material β-NaYF_4_ prepared using hydrothermal method is the current well-recognized substrate of the highest luminous efficiency^53^. Through comparing with standard card, it can find the compound material β-NaYF_4_:Ho^3+^@TiO_2_ not only involved all diffraction peaks of β-NaYF_4_, but also showed the characteristic diffraction peak of TiO_2_ when 2 θ = 25.4°, which was consistent with anatase standard card (JCPDS no. 21–1272). Therefore, it proved that the TiO_2_ included in photocatalyst prepared by sol-gel method was anatase-typed TiO_2_, which enjoyed higher photocatalysis effect than rutile type TiO_2_ and was more helpful in increasing the photocatalysis activity of the compound material^55^. Moreover, β-NaYF_4_:Ho^3+^@TiO_2_ prepared by such method will not change the crystal form of β-NaYF_4_, therefore it can be known such method has sound stability and reproducibility. Through comparing the XRD pattern between β-NaYF_4_:Ho^3+^@TiO_2_-rGO and the compound material, it can observe no occurrence of new peak value, and all peak values are either hexagonal hexagonal or anatase-typed TiO_2_. It can also be noted that the diffraction peak of rGO is not reflected in such XRD pattern, which we think may be due to its low proportion in compound material and its low concentration that is under the LOD of XRD. Therefore, other characterization methods are still needed to verify the existence of rGO.

In addition, it is worth noting that it can observe no diffraction peaks of Ho^3+^ from UC material, compound material, or ternary complex in above figure, which is either because Ho is not successfully doped in, or because it is exsited in other forms. The existence form of Ho is of vital significance to UC luminousmechanism, therefore it is needed to seek other characterization methods.

**Figure 2:**
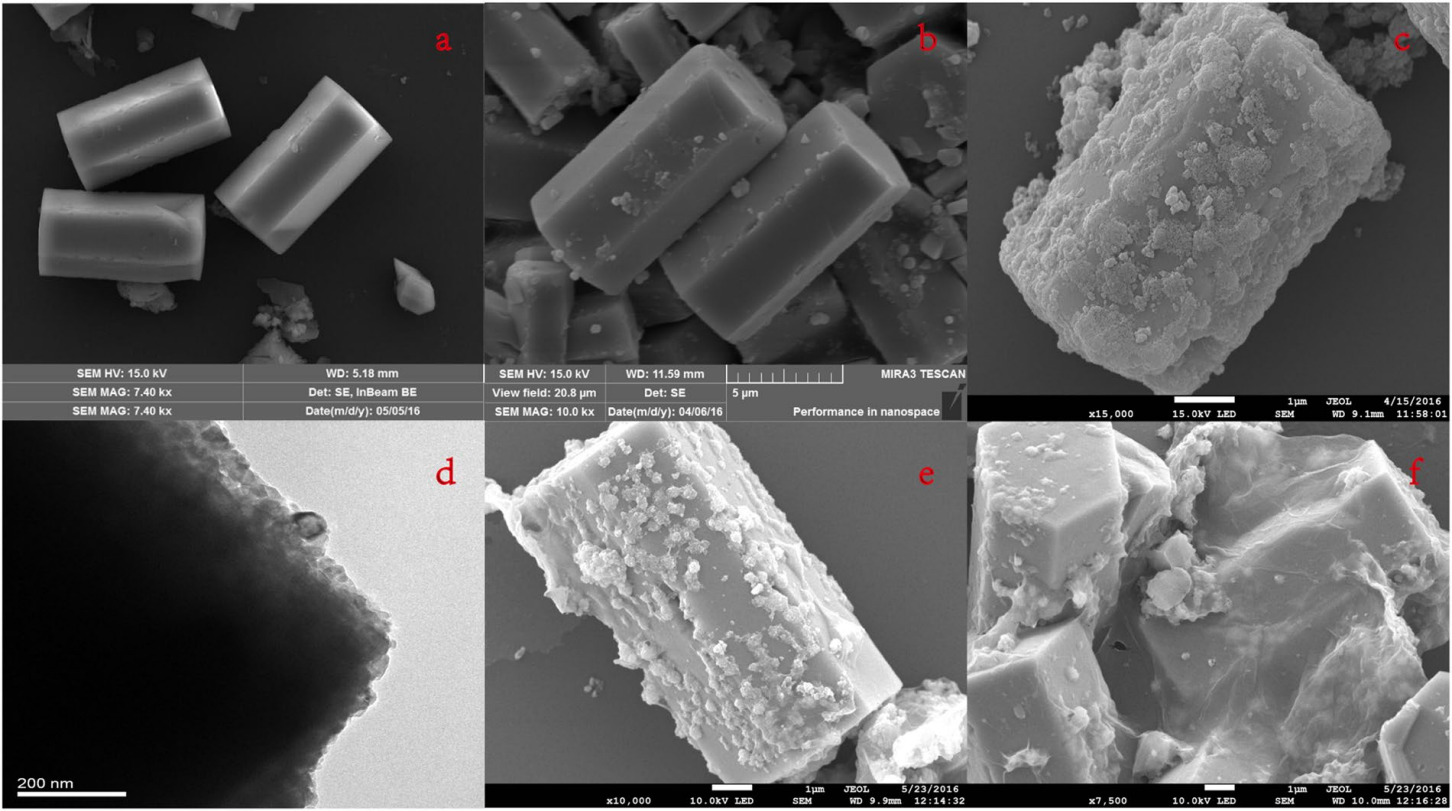
SEM images of (**a**,**b**) β-NaYF_4_:Ho^3+^, (**c**) β-NaYF_4_:Ho^3+^@TiO_2_, and (**e**,**f**) β-NaYF_4_:Ho^3+^@TiO_2_–rGO, (**d**) TEM images ofβ-NaYF_4_:Ho^3+^@TiO_2_.

To determine the micromorphologies and the presence of rGO these samples, we conducted SEM and TEM characterization for β-NaYF_4_:Ho^3+^, β-NaYF_4_:Ho^3+^@TiO_2_, andβ-NaYF_4_:Ho^3+^@TiO_2_-rGO, respectively, and the results are shown in Figure. 2. From Figure. 2a,b, it can be seen that the prepared UC materials are hexagonal prisms in uniform sizes, regular shapes, and with smooth surfaces. These hexagonal microcrystals have lengths of around 8 um and diameters of nearly 2.4 um, which is consistent with XRD characterization result. There is a close correlation between UC luminous efficiency and host material size. The larger the host material size is, the higher the luminous efficiency will be^56^. This indicates that the UC host material prepared by hydrothermal method is of sound luminous efficiency. After being compounded with TiO_2_ shell by sol-gel method, the UC material had not changed very much on its morphology, but still remain in shape of hexagonal prism. After coating, the surfaces become coarser (Figure. 2c), showing that the microcrystals are successfully coated with a TiO_2_ layer. The β-NaYF_4_:Ho^3+^ microcrystals are equably coated by the TiO_2_ shell. For further detailed structure analysis, the characterization of the β-NaYF_4_:Ho^3+^@TiO_2_ core-shell microcrystals was carried out by TEM. Figure 2d show enlarged image in which the β-NaYF_4_:Ho^3+^@TiO_2_ core-shell structure is clearly seen; the core of β-NaYF_4_:Ho^3+^ exhibits a dark color. These images confirm that the UC microcrystals are uniformly coated by a TiO_2_ layer. The average thickness of the TiO_2_ shells is about 50 nm. Core-shell structure model is beneficial to realizing high efficient energy transfer between UC material and photocatalyst. The SEM image of ternary complex β-NaYF_4_:Ho^3+^@TiO_2_-rGO in Figure. 2e,f shows that there are massive amounts of rGO lamellas deposited on the surface of compound material. There are small amount of deciduous TiO_2_ existing on the surfaces of some rGO lamellas; some rGO serve as substrates, on which compound materials are loaded; some rGO serve as carriers, which wrap the compound materials; The coupling between rGO and β-NaYF_4_:Ho^3+^@TiO_2_ is positive to the high efficient transferring of charge, and thus increasing the separation efficiency of photongenerated carriers.

### Composition and chemical states

**Figure 3:**
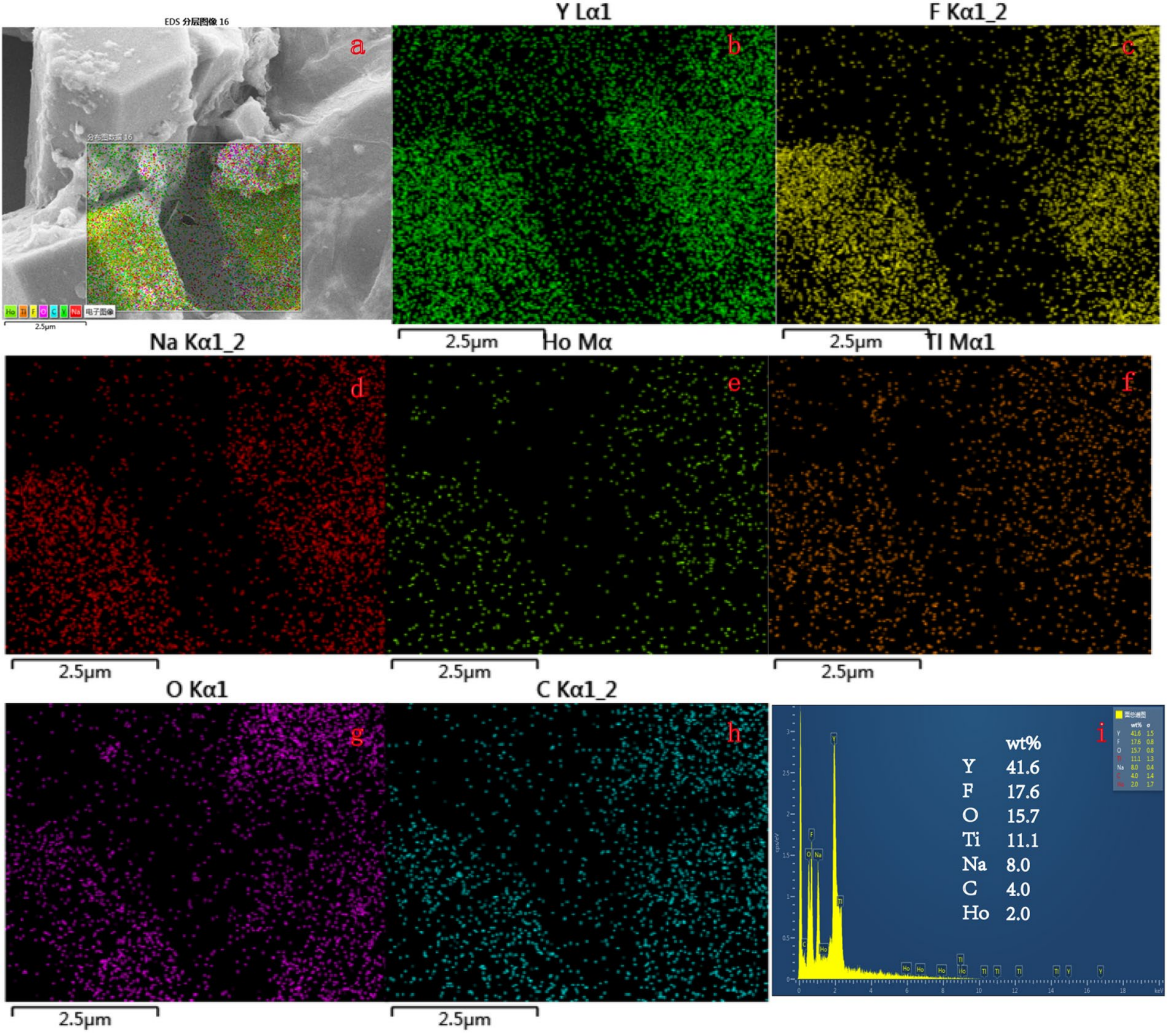
(**a**) SEM images ofβ-NaYF_4_:Ho^3+^@TiO_2_–rGO, (**b**–**h**) SEM elemental distribution mappings of Y, F, Na, Ho, Ti, O, and C. (**i**) EDS spectrum of β-NaYF_4_:Ho^3+^@TiO_2_–rGO.

Energy dispersive X-ray spectroscopy (EDS) was conducted to determine the element composition of β-NaYF_4_:Ho^3+^@TiO_2_-rGO and to further confirm the successful doping of Ho element. Results are shown in Figure. 3. According to the scan results of EDS surface of ternary compound material in Figure. 3b–h, it can observe the existences of Y, F, Na, Ho, Ti, O and C in ternary compound material β-NaYF_4_:Ho^3+^@TiO_2_-rGO, wherein C element is in homogeneous distribution, which indicates a sound dispersion effect of rGO. Figure 3i presents the mass percent of each element. However, EDS can only characterize the existence of in prepared sample, rather than charaterizing the existence form of Ho.

**Figure 4:**
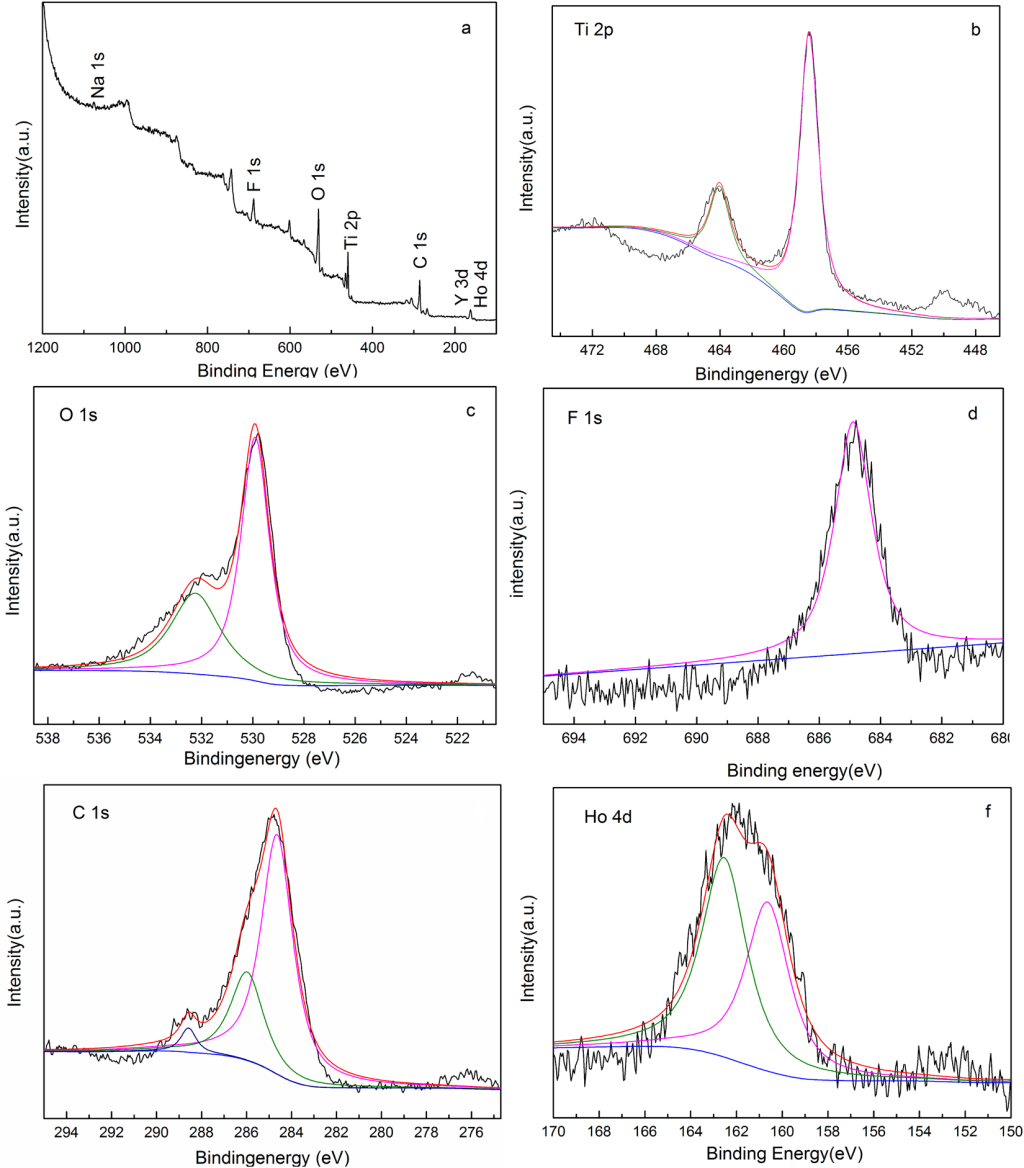
High-resolution XPS analyses of the β-NaYF_4_:Ho^3+^@TiO_2_-rGO microcrystals: (**a**) Wide spectrum, (**b**) Ti 2p, (**c**) O 1 s, (**d**) F 1 s, (**e**)C 1 s, (**f**) Ho 4d.

XPS characterization method was used to determine the chemical states of elements on the surface of β-NaYF_4_:Ho^3+^@TiO_2_-rGO as shown in Figure. 4. According to the full spectrum image in Figure. 4a, it shows that such sample contains Ti, O, Na, Y, F, Ho and C elements. C 1 s peak (284.1 eV) is the spectrum internal reference. Figure 4b shows that the Ti2p photoemission peak of compound material consists of two sub-peaks, with binding energies of 458.3 eV and 464.1 eV, corresponds to Ti 2p_3/2_ and Ti 2p_1/2_, respectively, which is consistent with the XPS spectrum of TiO_2_ as described^57^. As shown by the O1s in Figure. 4c, there is at least one type of oxygen in the compound material. The binding energies of two peaks are 529.9 eV and 532.3 eV, which are corresponded to the characteristics of Ti–O–Ti and H–O, respectively. The element F displays one characteristic peak at 684.3 eV because of the core level of F1s (see Figure. 4d). The C1s XPS spectrum shows two characteristic peaks, corresponding to oxygenated ring C bonds (284.7 eV for C-C, C=C and C-H,286.2 eV for C-O, and 288.6 eV for the C=O bond). These results indicate that there exist abundant oxygen-containing functional groups onrGO surface. However, in the C 1 s XPS spectra of β-NaYF_4_:Ho^3+^@TiO_2_-rGOas shown in Fig. 4e, the relative intensities of the three components associated with C-O/C=O bonds decrease significantly, indicating that some of the oxygen functional groups were reduced during the chemical reduction process^58,59^. Doped elements can also be detected by XPS. Figure 4f represents the characteristic peak of Ho^3+^ at binding energy of 159.5 eV and 161.1 eV, respectively. XPS characterization results show that the Ho element in the sample exists in the form of Ho^3+^ and has been successfully doped into the crystal lattice of the host material β-NaYF_4_.

**Figure 5:**
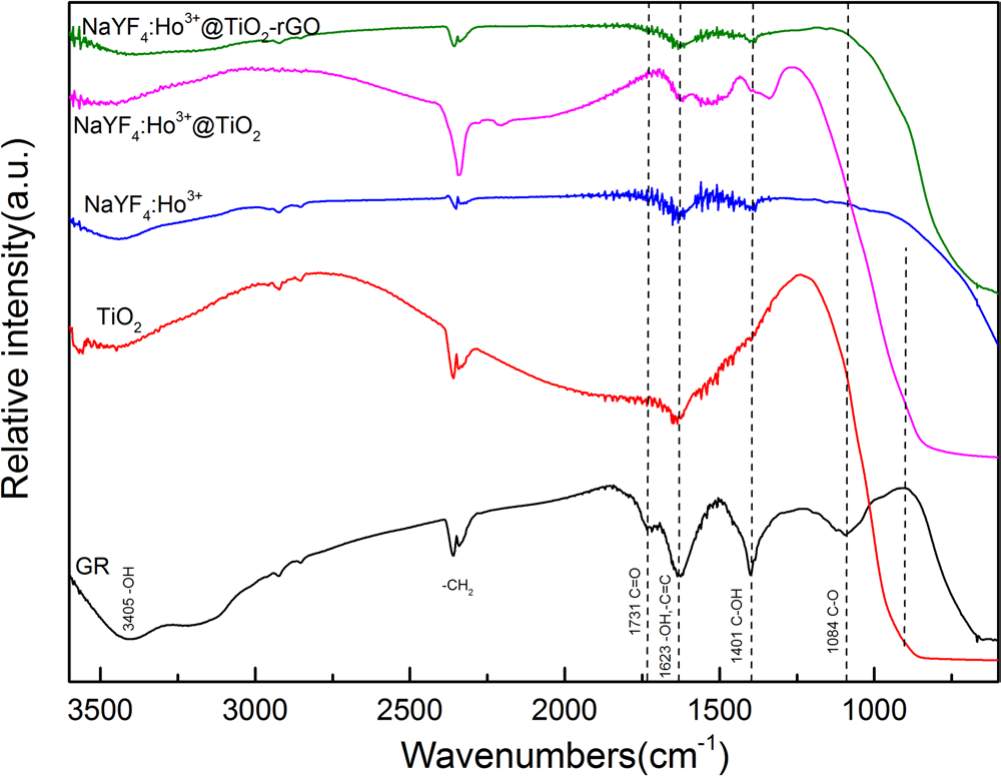
FTIR spectra of GO, TiO_2_, β-NaYF_4_:Ho^3+^, β-NaYF_4_:Ho^3+^@TiO_2_, and β-NaYF_4_:Ho^3+^@TiO_2_-rGO, respectively.

To further confirm the reduction of GO, we conducted FTIR analysis for the samples, and the results are shown in Figure. 5. It shows that GO contains abundant oxygen containing functional groups. The strong peak occurred at 1731 cm^−1^ is mainly caused by the tensile vibration of C=O in carboxyl functional groups, while the strong peak at around 1623 cm^−1^ is mainly caused due to the lack of oxidized C=C structure in graphite structure. In addition, the occurrence of peak at 1401 cm^−1^ is due to the oxidized C-OH on the surface. The occurrences of peaks at 3405 cm^−1^ and 1048 cm^−1^ are due to the vibrations of OH and C-O^60^, respectively. Through comparing β-NaYF_4_:Ho^3+^@TiO_2_-rGO and GO, it can find that C-O and C=O, at 1084 cm^−1^ and 1731 cm^−1^ respectively, are almost disappeared, which indicates that some carboxy groups are differentially reduced according to different conditions. The functional groups at 1623 cm^−1^ and 1401 cm^−1^ are also significantly reduced. Results show that the GO can be effectively reduced into rGO using hydrothermal method, which is consistent with XPS result. β-NaYF_4_:Ho^3+^@TiO_2_-rGO displays a strong and wide absorption peak, which is proved to be a combination peak caused by the stretching vibration of Ti-O-Ti and Ti-O-C^61^. Moreover the existence of Ti-O-C bond indicates existence of chemical bond force between rGO and TiO_2_, and such chemical bond is beneficial to the red shift of absorbed wavelength^49^, so as to better take the advantage of solar photocatalysis.

### Photoluminescence properties

**Figure 6:**
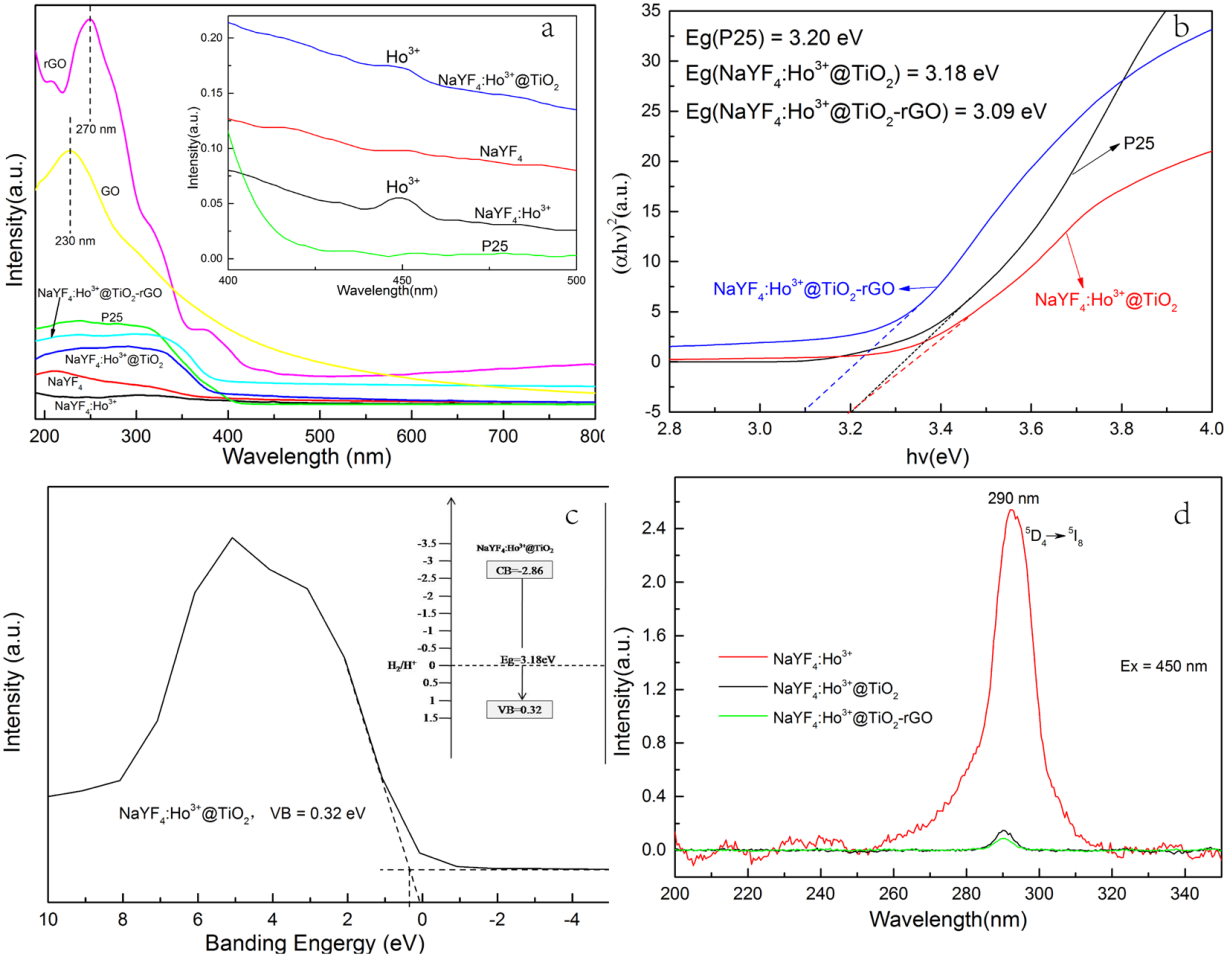
(**a**) The UV-Vis absorbance spectra of the P25, β-NaYF_4_, β-NaYF_4_:Ho^3+^, β-NaYF_4_:Ho^3+^@TiO, β-NaYF_4_:Ho^3+^@TiO_2_-rGO, GO, and the RGO prepared by hydrothermal method under the same conditions asβ-NaYF_4_:Ho^3+^@TiO_2_-rGO; inset: the enlarged spectra ranging from 400 nm to 500 nm. (**b**) The plot for bandgap energy Eg. (**c**) VB XPS; inset: schematic illustration of the band gap structures. (**d**) Photoluminescence (PL) spectra of samples under 450 nm excitations.

Figure 6a shows the UV-Vis diffuse reflection spectrum of ternary compound material β-NaYF_4_:Ho^3+^@TiO_2_-rGO. Results show that the UV-Vis diffuse reflection spectrum of β-NaYF_4_:Ho^3+^@TiO_2_-rGO has two distinguished features as compared with β-NaYF_4_:Ho^3+^@TiO_2_, β-NaYF_4_:Ho^3+^, β-NaYF_4_, and P25, wherein one is the overall improvement of Vis light absorption property, and the other one is the red shift of absorption cross section. Both features are positive to increasing the photocatalytic activity^7^. In addition, we observed that β-NaYF_4_:Ho^3+^ displayed three weak absorption peaks at 450 nm, 537 nm, and 642 nm, respectively, while β-NaYF_4_ did not show absorption peak. This indicates the UC material excited ion Ho^3+^ is of vital significance to luminescence. On the whole, the absorption peak at 450 nm is relatively stronger. As we know that the stronger the light-absorbing capacity of the absorption peak is, the more suitable the absorption peak will be serving as excitation wavelength. Therefore the absorption peak at 450 nm was finally selected as the excitation wavelength for the luminescence spectrum of UC material β-NaYF_4_:Ho^3+^.In the absorption spectrum of GO, it shows the absorption peaks of GO at 230 nm (π-π* transitions of C=C bonds)^62^ referred to pure GO films shown in Figure. 6a. rGO has a characteristic absorption peak at 270 nm, compared with GO 40 nm red shift of absorption spectrum, the overall absorption intensity increases, indicating that GO in hydrothermal conditions reduction. The carbon atoms of sp^3^ hybridized into sp^2^ hybrid structure, improved GR was largeπelectron conjugated structure^63^. sUV-Vis characterization results show that the rGO was successfully prepared by hydrothermal synthesis.

The bandgaps of P25, β-NaYF_4_:Ho^3+^@TiO_2_, and β-NaYF_4_:Ho^3+^@TiO_2_-rGO can be calculated using Tauc’s formula, and the calculation results are shown in Figure. 6b. The bandgap of P25 is 3.20 eV (387.5 nm), the bandgap of β-NaYF_4_:Ho^3+^@TiO_2_ is 3.18 eV (389.9 nm), and the bandgap of β-NaYF_4_:Ho^3+^@TiO_2_-rGO is 3.09 eV (401.3 nm). From the results, it can conclude that the UC materialβ-NaYF_4_:Ho^3+^ microcrystal is of limited influence to the bandgap of TiO_2_, while the introduced rGO is of larger influence to its absorption cross section, such as reducing its bandgap and leading to the red shift of absorbed wavelength. From Figure. 6c, it can be seen that the VB edges ofβ-NaYF_4_:Ho^3+^@TiO_2_ are estimated to be 0.32 eV. According to the bandgap and the valence band (VB) position of the samples, we can draw the bandgap structures as displayed in Figure. 6c inset, from which it can be clearly seen that the conduction band (CB) and VB position of β-NaYF_4_:Ho^3+^@TiO_2_.

Figure 6d shows the UC Luminescence spectra of β-NaYF_4_:Ho^3+^, β-NaYF_4_:Ho^3+^@TiO_2_, and β-NaYF_4_:Ho^3+^@TiO_2_-rGO respectively under 450 nm excitation. It can be seen that under 450 nm excitation, there is a emission peak at 290 nm in ultraviolet region, which corresponds to the radiative transition of Ho^3+^ from ^5^D_4_ to ^5^I_8_. After being doped with TiO_2_, the emission peak wavelength location of composite material of β-NaYF_4_:Ho^3+^ has not been changed, which indicates that the UC luminance properties remain unchanged after compounded with TiO_2_. In addition, we can observe that β-NaYF4:Ho^3+^@TiO_2_ shows a fairly weak luminous intensity at 290 nm under the 450 nm excitation, wherein the emission peak is almost disappeared. This may be because the UV-light emitted from β-NaYF_4_:Ho^3+^was absorbed by the TiO_2_ wrapped on the surface, so that the phenomenon of photocatalysis is resulted. As it is well known that rGO is an effective material which is of high ability in absorbing light^64^. For rGO-assisted β-NaYF_4_:Ho^3+^@TiO_2_, there were less light emitted from the system, while more irradiated lights were absorbed in. It is reasonable that stronger irradiated light will induce higher intensity of the converted light. Hence, the low emission intensity detected here can be ascribed to the fact that most of the converted lights were absorbed by the composite with the help of rGO.

### Ramna spectra

**Figure 7:**
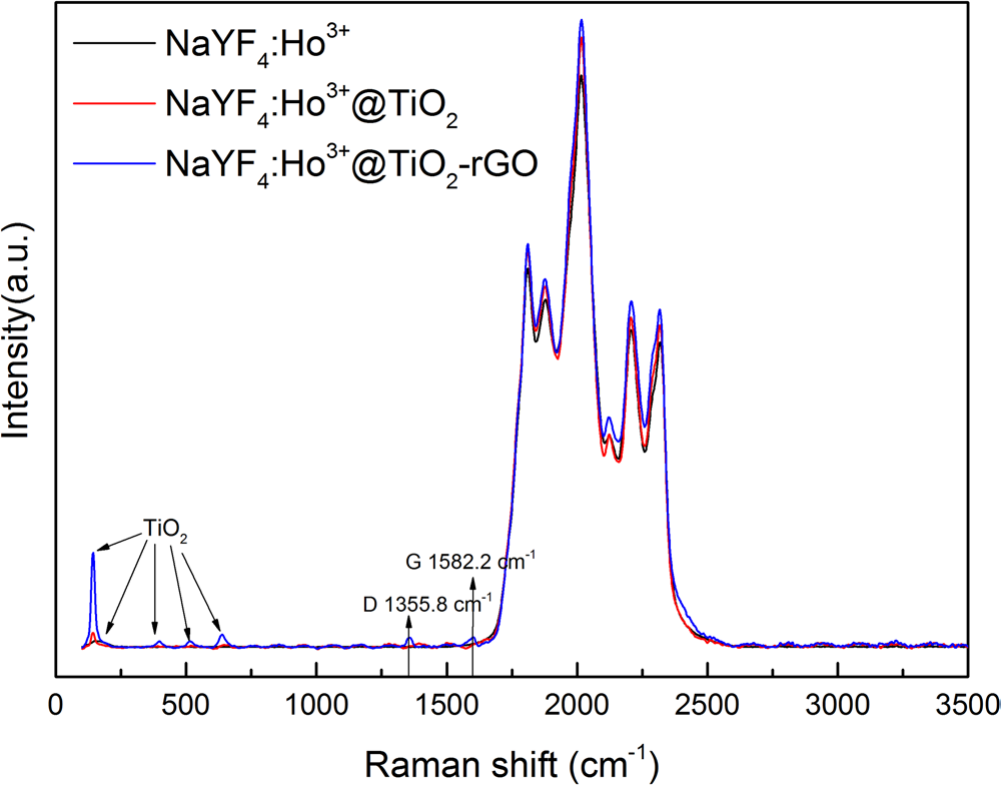
Raman spectra of NaYF_4_:Ho^3+^, NaYF_4_:Ho^3+^@TiO_2_, and NaYF_4_:Ho^3+^@TiO_2_-rGO composite.

The Raman spectra of NaYF_4_:Ho^3+^, NaYF_4_:Ho^3+^@TiO_2_, and NaYF_4_:Ho^3+^@TiO_2_-rGO composite are presented in Figure. 7. The Raman spectrum of rGO displayed two prominent peaks at 1355.8 and 1582.2 cm^−1^, corresponding to the well-documented D and G bands, respectively^65^. G band is Raman active for sp^2^ hybridized carbon-based material, while D band is activated only if defects participate the double resonance Raman scattering near K point of Brillouin zone^63^. The specific vibration modes are located around 140, 399, 517, and 639 cm^−1^, indicating the presence of the anatase phase in all of these samples^66^. The intensity ratio of D band to G band (I_D_/I_G_) is correlative with the average size of sp^2^ domains^67^. Higher I_D_/I_G_ ratio means the smaller size of sp^2^ domains. From Figure. 6, the I_D_/I_G_ ratios for NaYF_4_:Ho^3+^@TiO_2_-rGO were1.04, further confirmation theNaYF_4_:Ho^3+^@TiO_2_-rGO composite further confirmed the formation of rGO sheets^63^. Raman spectroscopy is one of the most common, rapid, non-destructive and high-resolution techniques for characterizing carbon materials^67^. Previous studies have shown that certain Raman peaks change sensitively with the number of GR layers (n)^68^. This discovery that exhibits Raman fingerprints for single-layer, bilayer, and few-layer GR can be used as identification. The position of the G (1582.2 cm^−1^) peak of the Raman spectrum was deduced from previous studies that the number of rGO layers was within six layers^67^.

### Photoelectrochemistry measurements

**Figure 8:**
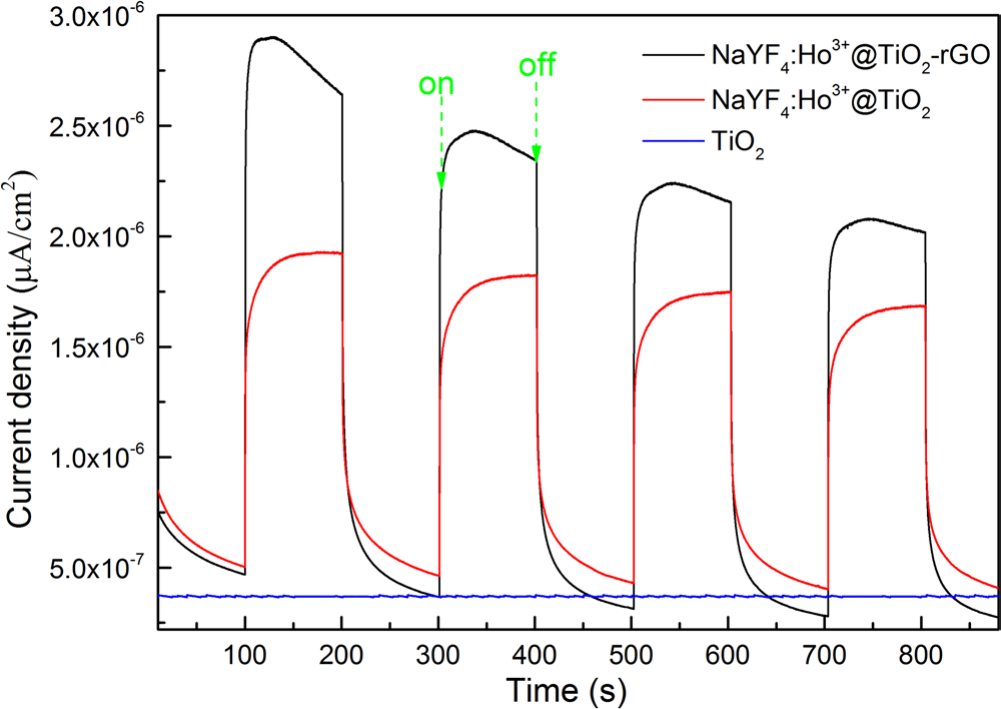
Transient photocurrent densities of the as-prepared samples.

Photoelectrochemical measurements were performed to investigate the excitation, separation, transfer, and recombination of photoinduced charge carriers^69^. Figure 8 shows the characterization results of photocurrents of electrode TiO_2_, β-NaYF_4_:Ho^3+^@TiO_2_, and β-NaYF_4_:Ho^3+^@TiO_2_-rGO, based on which it can deduce the electron inteaction between rGO and β-NaYF_4_:Ho^3+^@TiO_2_. From the Figure. 8 we can see that steady and transient photocurrents can be obtain during the process of cyclically opening and closing Vis light irradiation. Enjoying a large bandgap, the TiO_2_ nanoparticle is of no influence to Vis light, and nearly no photocurrents were resulted. However regarding composite material β-NaYF_4_:Ho^3+^@TiO_2_, the UC material within first absorbed Vis light and converts it into UV light, and then excited TiO_2_ to produce electron-hole pairs, so that photocurrents were produced. It is worth noting that the photocurrent resulted from electrode β-NaYF_4_:Ho^3+^@TiO_2_-rGO has stronger intensity as compared with photocurrent resulted from β-NaYF_4_:Ho^3+^@TiO_2_. This is owe to rGO’s excellent conductivity that led to the effective separation of electron-hole pairs. The effective separation of electron-hole pairs is a key factor for the enhancement of photocatalysis activity of β-NaYF_4_:Ho^3+^@TiO_2_-rGO. The results above are consistent with PL analysis results.

### Photocatalytic activity and recyclability

**Figure 9:**
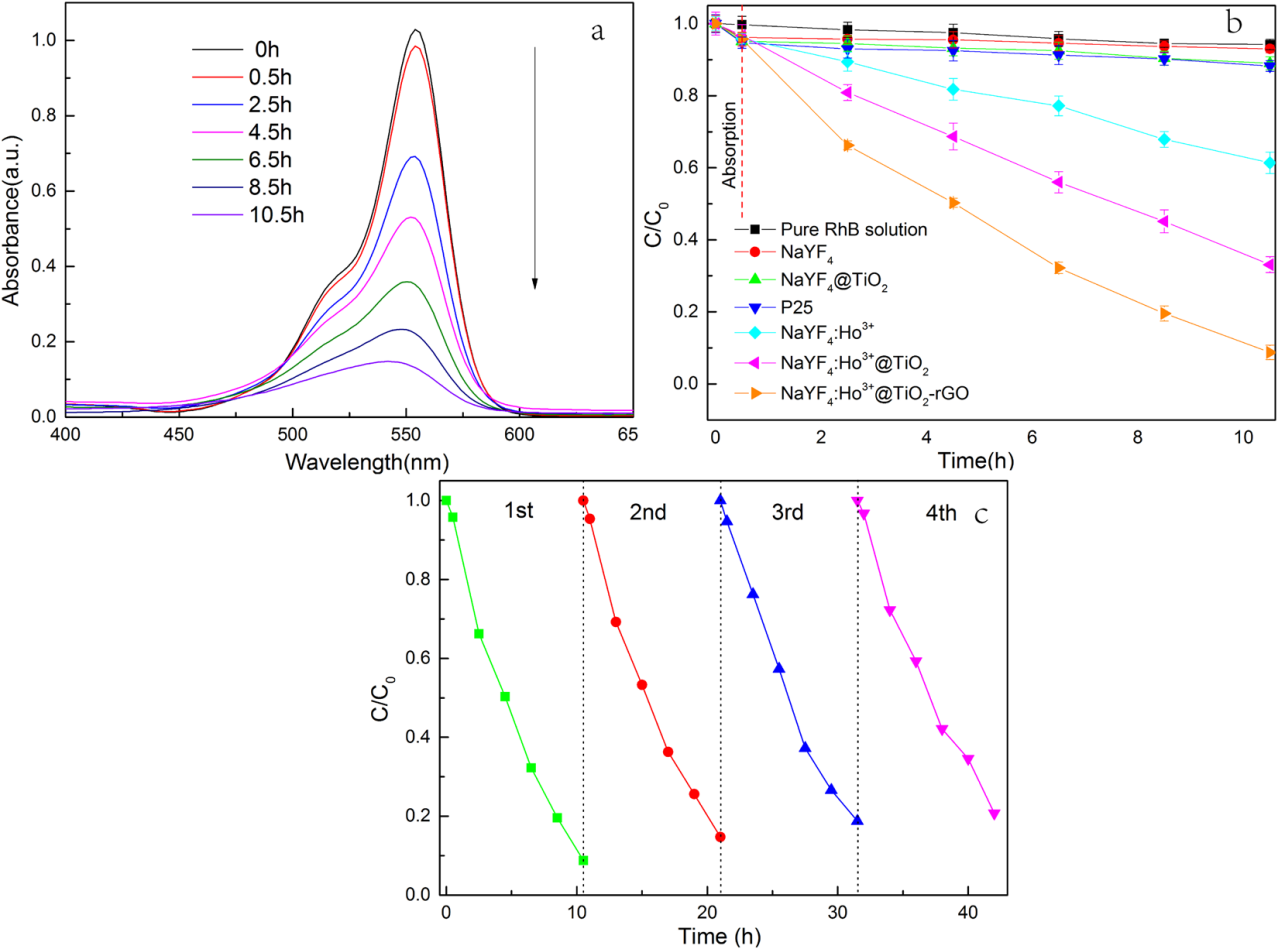
(**a**) Time-dependent UV-Vis absorption spectroscopy of an RhB solution degraded by β-NaYF_4_:Ho^3+^@TiO_2_-rGO (**b**) Time-dependent plot of the photodegradation of a RhB solution by different catalysts upon irradiation by a Xe lamp (500 W) with a UV cutoff filter (λ > 400 nm). (**c**) Cyclic run of the photocatalytic degradation of RhB using β-NaYF_4_:Ho^3+^@TiO_2_-rGO.

Based on RhB as target degradation product, we researched the photocatalytic activity of β-NaYF_4_:Ho^3+^@TiO_2_-rGO to Vis light. We used 500 Xe lamp coupled with 420 nm cut-off filter as the light source. The time-dependent absorption spectrogram of RhB is shown in Figure. 9a, from which we can see that RhB shows a gradual decreasing absorbance at 554 nm with the extension of time. This indicates β-NaYF_4_:Ho^3+^@TiO_2_-rGO is of sound degradation effect to RhB under Vis light irradiation.

**Figure 10:**
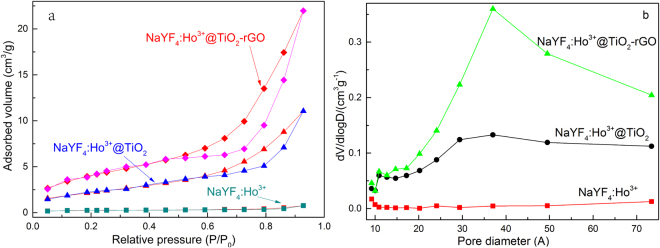
N_2_ adsorption-desorption isotherms (**a**) and pore size distribution curves (**b**) of β-NaYF_4_:Ho^3+^, β-NaYF_4_:Ho^3+^@TiO_2_, and β-NaYF_4_:Ho^3+^@TiO_2_-rGO.

Figure 9b shows the variation curve of C/C_0_ with the increase of optical radiation time, wherein C_0_ and C respectively represent the initial concentration of RhB solution and the concentration of RhB after certain period of irradiation time. Results show that photocatalyst β-NaYF_4_:Ho^3+^@TiO_2_-rGO can reach decomposition rate of RhB as 92% after 10 h of Xe lamp irradiation, which is increased by 25% as compared to the decomposition rate of RhB using β-NaYF_4_:Ho^3+^@TiO_2_ after 10 h of Xe lamp irradiation. Theβ-NaYF_4_:Ho^3+^@TiO_2_-rGO exhibited the best photocatalytic efficiency, which was because its larger BET surface area and pore volume (Figure. 10 and Table 1) are beneficial for theβ-NaYF_4_:Ho^3+^@TiO_2_-rGO composite contacting with organic contaminants, and thus the photocatalytic performance can be enhanced after loading of the rGO sheets. In addition, the rGO facilitated the transport of electrons photogenerated in the TiO_2_, and therefore led to efficient separation of photogenerated carriers in the coupledβ-NaYF_4_:Ho^3+^@TiO_2_-rGO system. Moreover, the photocatalytic degradation of RhB was closely dependent on the Vis light adsorption ability of TiO_2_, and the loading of the rGO shortened the bandgap of TiO_2_. This explains why there was an increased absorptionof Vis light on TiO_2_, and thus why an increased decomposition rate of RhB was resulted in. To eliminate external factors to the degradation effect of phototacatlysis, we conducted a series of comparative tests. We found that the self-decomposition rate of RhB after 10 h of Xe lamp irradiation was only 6%, which indicates the thermal radiation of xenon lamp is of limited influence. By using UV catalyst P25, the decomposition rate of RhB after 10 h of Xe lamp irradiation was only 12%, wherein half of the decomposition rate was ascribed to adsorption degradation. To our surprise, the UC materialβ-NaYF_4_:Ho^3+^ reached the decomposition rate of RhB as high as 37% after 10 h of Xe lamp irradiation, which was higher than those of β-NaYF_4_, P25, and β-NaYF_4_@TiO_2_. This is mainly because the non-radiative transition of activator Ho^3+^ in UC material resulted in the UV light of short wavelength (290 nm) which is of higher thermal energy and photosensitization effect, so that RhB can be decomposed more effectively^70^. In addition, due to the absence of doping with Ho^3+^, the β-NaYF_4_ and β-NaYF_4_@TiO_2_ after 10 h of Xe lamp irradiation can achieve decomposition rate of RhB as 7% and 11%, respectively, which were far below the levels after doping with Ho^3+^. This indicates that suitable activator must be selected to induce photon transition before realizing effective UC luminescence.

**Table 1:** Characteristics obtained from nitrogen desorption isotherms.

Sample	Mean pore size(nm)	Pore volume(cm^3^g^−1^)	Surface area(m^2^g^−1^)
**NaYF**_**4**_ **:Ho** ^**3+**^	3.5123	0.001069	0.8938
**NaYF**_**4**_ **:Ho** ^**3+**^ **@TiO**_**2**_	3.5915	0.016921	8.3884
**NaYF**_**4**_ **:Ho** ^**3+**^ **@TiO**_**2**_ **-rGO**	3.9655	0.032805	16.7511

Photocatalyst should remain unchanged photochemical stability and durability after repeated irradiation, which is of vital significance in practical application. Figure 9c shows the photocatalystic activity of β-NaYF_4_:Ho^3+^@TiO_2_-rGO after four repeated irradiations. From this figure we can see that after four repeated irradiations, the photocatalytic activity of the sample is insignificantly decreased from 92% to 81% and then tends to be stable. This reveals that the composite photocatalyst is of relatively better stability and reproducibility.

### Photocatalyticradical and mechanisms analysis

**Figure 11:**
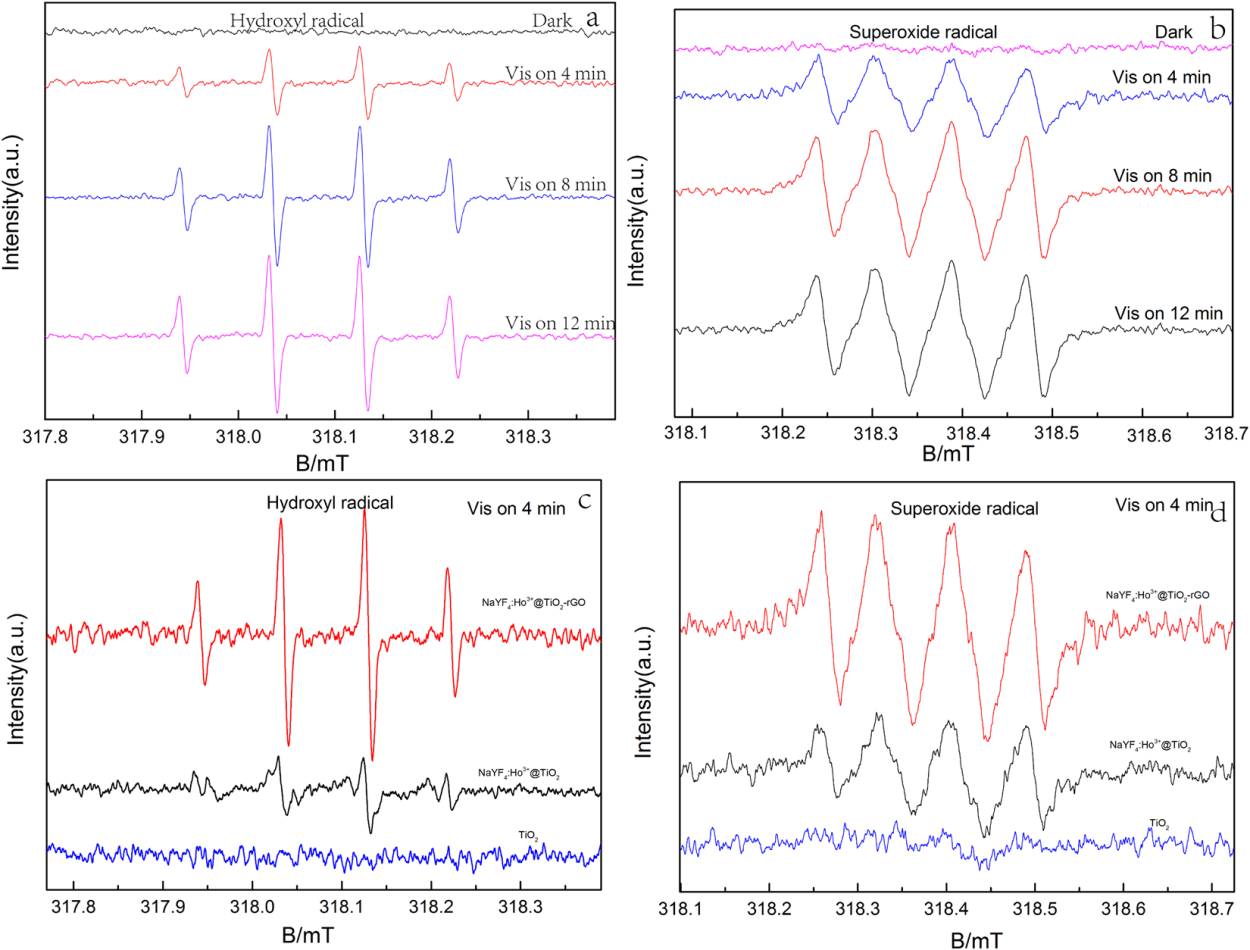
DMPO spin-trapping ESR spectra of β-NaYF4:Ho^3+^@TiO2-rGO in methanol dispersion for OH (**a**) and in aqueous dispersion for·O2^−^ (**b**); (**c**) superoxide radical, (**d**) hydroxyl radicals of TiO2, β-NaYF4:Ho^3+^, and β-NaYF4:Ho^3+^@TiO2-rGO after four min of Vis light irradiation.

DMPO-ESR spin trapping spectrum was used to detect ·OH and ·O_2_^−^ in solution^71,72^. As shown in Figure. 11a,b, under Vis light irradiation, ·OH and ·O_2_^−^ in β-NaYF_4_:Ho^3+^@TiO_2_-rGO can be successfully detected. Moreover, with the extension of time, the signals of both free radicals are significantly increased, which indicates the accumulation effect of both free radicals as time goes on. To illustrate the influence of the introduction of rGO to the system, we compared the signals of ·OH and ·O_2_^−^ of TiO_2_, β-NaYF_4_:Ho^3+^@TiO_2_, and β-NaYF_4_:Ho^3+^@TiO_2_-rGO at the same time under Vis light irradiation, shown in Fig. 11c,d. After 4 min of Vis light irradiation, there are significant differences on the two free radicals of TiO_2_, β-NaYF_4_:Ho^3+^@TiO_2_, and β-NaYF_4_:Ho^3+^@TiO_2_-rGO. As for TiO_2_, the Vis light is not enough to excite TiO_2_, so that the signals of both free radicals can hardly be detected. Since β-NaYF_4_:Ho^3+^@TiO_2_ is capable of converting Vis light into UV light, and excite TiO_2_ to result separation of electron-hole pairs, it can detect strong signals of ·OH and ·O_2_^−^. After introducing rGO, i.e. in β-NaYF_4_:Ho^3+^@TiO_2_-rGO system, the signals of both radicals are significantly increased, wherein the signals of ·OH and ·O_2_^−^ are 3.2-fold and 2.1-fold of those in β-NaYF_4_:Ho^3+^@TiO_2_ system. This result indicates that β-NaYF_4_:Ho^3+^@TiO_2_-rGO can generate more free radicals to take part in photocatalytic reaction, so as to improve the photocatalytic activity. Owe to the super conductive performance of rGO, larger amounts of free radicals can be produced, which can effectively separate electron-hole pairs and extend the lifetime of charge carrier.

### 3D fluorescence spectra

**Figure 12:**
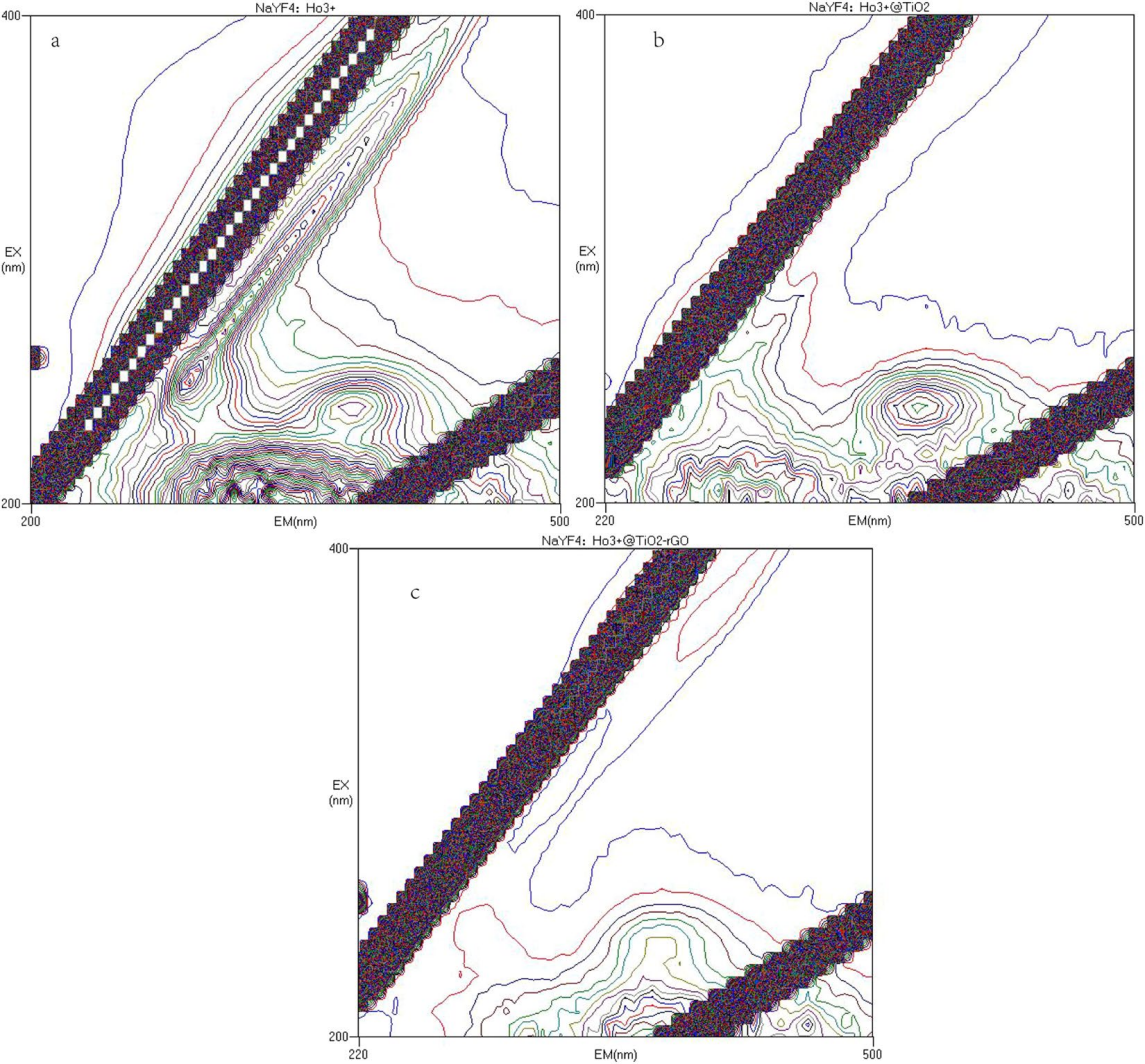
3D fluorescence spectra of NaYF4:Ho^3+^ (**a**), NaYF4:Ho^3+^@TiO2 (**b**), and NaYF4:Ho^3+^@TiO2-rGO composite (**c**).

Photoluminescence (PL) spectra reflect the migration, transfer, and recombination processes of the electronhole pairs^73,74^. Figure 12 shows 3D fluorescence spectra β-NaYF_4_:Ho^3+^, β-NaYF_4_:Ho^3+^@TiO_2_, and β-NaYF_4_:Ho^3+^@TiO_2_-rGO composite. The plots show that UC and catalysts have a maximum fluorescence peak near (λ_ex_, λ_em_) = (205 nm, 315 nm(335 nm)), (λ_ex_, λ_em_) = (240 nm, 315 nm), and (λ_ex_, λ_em_) = (205 nm, 315 nm), which is attributed to the recombination of holes and electrons across the band gap of β-NaYF_4_:Ho^3+^. The β-NaYF_4_:Ho^3+^@TiO_2_ composites absorb slightly weakly than β-NaYF_4_:Ho^3+^, which implies that the recombination of photogenerated electrons and holes is less in the β-NaYF_4_:Ho^3+^@TiO_2_ composites. The β-NaYF_4_:Ho^3+^@TiO_2_-rGO composites absorb more weakly than β-NaYF_4_:Ho^3+^ and β-NaYF_4_:Ho^3+^@TiO_2_, which implies that the recombination of photogenerated electrons and holes is much less in the β-NaYF_4_:Ho^3+^@TiO_2_-rGO. The results above are consistent with Photoelectrochemical measurements analysis results.

**Figure 13:**
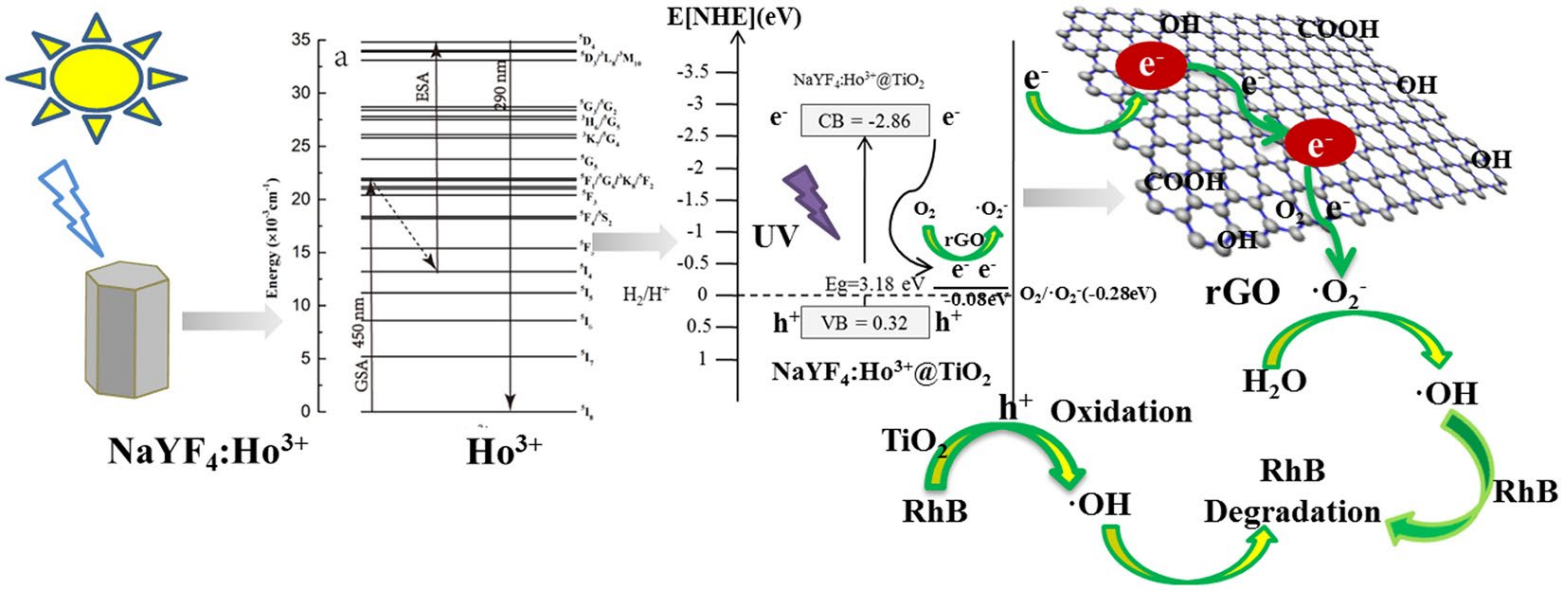
Photocatalytic reaction mechanism of β-NaYF4:Ho^3+^@TiO2-rGO.

A possible reaction process is proposed in Figure. 13, which can be summarized in the equations below. The β-NaYF_4_:Ho^3+^UC absorbs visible light and emits UV light (R1). The detailed process can be described as below. First Ho^3+^ jumps from ground state ^5^I_8_ energy level to excited state ^5^F_1_ (^5^I_8_ → ^5^F_1_) energy level via Ground State Absorption (GSA) under the excitation of 450 nm light source, meanwhile it jumps via nonradiative cross relaxation back to excited state ^5^I_4_ energy level (^5^F_1_ → ^5^I_4_), rather than back to excited state ^5^I_6_ energy level. In the second stage, the Ho^3+^, which locates on excited state ^5^I_4_ energy level, absorbs the photon of the same energy, and then directly jumps via ESA onto excited state ^5^D_4_ energy level (^5^I_4_ → ^5^D_4_). Finally, Ho^3+^ jumps from highly excited level ^5^D_4_ back to ground state ^5^I_8_ (^5^D_4_ → ^5^I_8_), while emitting 288 nm UV light, so that the two-photon UC luminescence mechanism is completed. Through UV excitation, electron-hole pairs are generated on the TiO_2_ surface (R2), which is followed by rapid transfer of photogenerated electrons to rGO sheets via percolation mechanism (R4). The excited electrons on the TiO_2_ surface react with the absorbed oxygen, resulting in the formation of ·O_2_^−^ or HO_2_·. After that, it can form hydrogen peroxide (H_2_O_2_; R5) through combining H^+^ with ·O_2_^−^ or HO_2_·. H_2_O_2_ reacts with the superoxide radical anion (·O_2_^−^), and then reduces it into a hydroxyl radical (·OH; R7). The photogenerated holes react with H_2_O, resulting in the formation of hydroxyl radicals (·OH; R3). The conduction band of TiO_2_ is located above the RhB redox potential, which allows TiO_2_ to be catalytically active. Therefore, these reactive oxygen species (i.e., ·OH, ·O_2_^−^, and H_2_O_2_), especially ·OH, can oxidize the organic molecules and perform photocatalysis (R7). The entire sequence is summarized as below:

1 $${{\rm{NaYF}}}_{4}:{{\rm{Ho}}}^{3+}+\,\mathrm{visible}\,\mathrm{light}\,\to {{\rm{NaYF}}}_{4}:{{\rm{Ho}}}^{3+}+\,\mathrm{UV}\,$$

2 $${{\rm{TiO}}}_{2}+\,{\rm{UV}}\to {{\rm{TiO}}}_{2}{({\rm{h}}}^{+}+{{\rm{e}}}^{-})$$

3 $${{\rm{H}}}_{2}{\rm{O}}+{{\rm{TiO}}}_{2}{({\rm{h}}}^{+})\to \cdot {\rm{OH}}+{{\rm{H}}}^{+}$$

4 $${{\rm{TiO}}}_{2}{({\rm{e}}}^{-})+{\rm{rGO}}\to {{\rm{TiO}}}_{2}+{\mathrm{rGO}({\rm{e}}}^{-})$$

5 $${{\rm{O}}}_{2}+{{\rm{H}}}^{+}+{\rm{rGO}}\,{({\rm{e}}}^{-})\to \cdot {{\rm{O}}}_{2}^{-}+{{\rm{H}}}_{2}{{\rm{O}}}_{2}$$

6 $$\cdot {{\rm{O}}}_{2}^{-}+{{\rm{H}}}_{2}{{\rm{O}}}_{2}\to \cdot {\rm{OH}}+{{\rm{OH}}}^{-}+{{\rm{O}}}_{2}$$

7 $$\cdot {\rm{OH}}\,(\mathrm{or}\cdot {{\rm{O}}}_{2}^{-})+{\rm{RhB}}\to \mathrm{degradation}\,\mathrm{products}$$

## Conclusions

In summary, we have successfully prepared β-NaYF_4_:Ho^3+^@TiO_2_-rGO composites as an advanced Vis-driven photocatalyst by using a simple hydrothermal method. We demonstrated a new strategy by integrating the Vis-to-UV UC property of β-NaYF_4_:Ho^3+^ with the excellent electrical properties of GR to enhance the photocatalytic efficiency of TiO_2_. Photocatalytic properties of the β-NaYF_4_:Ho^3+^@TiO_2_-rGO composites were evaluated by the degradation of an RhB solution. The enhanced photocatalytic activity was associated with the large extended photoresponsive range, great adsorptivity of dyes and high electron–hole separation efficiency due to the synergetic interactions among TiO_2_, GR and UC material. This work is expected to promote practical applications of photocatalysts under solar irradiation in the hope of addressing various environmental issues.

## Experimental Section

### Materials and reagents

All chemicals were used as received without further purification. Y_2_O_3_ (99.999% purity), Ho_2_O_3_(>99.9% purity) and GO solution(>99.85% purity) were of high purity. All other chemicals, including NaF, ethanol, ethylenediaminetetraacetic acid (EDTA), tetra-n-butyl titanate (TBOT), and polyvinylpyrrolidone K-30 (PVP) were in analytical grade. Y_2_O_3_, NaF, TBOT, and PVP were purchased from Chengdu Kelong Chemical Co. Ltd. (Sichuan, China). Ho_2_O_3_ was obtained from Shanghai Tongna Environmental Protection Co. Ltd. (China). EDTA was purchased from Chongqing Boyi Chemical Co. Ltd. (China). HNO_3_ and ethanol were obtained from Chongqing Chuandong Chemical Group Co. Ltd. (China).GO solutionwas obtained from Shanghai HuaYiCo. Ltd. (China).

### Synthesis of NaYF_4_:Ho^3+^@TiO_2_–rGOternarycomposites

The NaYF_4_:Ho^3+^@TiO_2_ core-shell microcrystals were synthesized by referring preliminary work^35^. UV-irradiation of theNaYF_4_:Ho^3+^@TiO_2_-graphene oxide samples was performed using a hydrothermal method. Firstly, 2 mg/mL GO (2.5 mL) was ultrasonicated in 100 mL of anhydrous ethanol solution till being well dispersed; after that, 0.1 g of NaYF_4_:Ho^3+^@TiO_2_was added into the above GO solution and then keep vigorous stirring for 1 h. The resulting mixture was transferred into a 100 mL stainless Teflon-lined autoclave filled with deionized water up to 80% of its capacity. The autoclave was tightly sealed and heated at 130 °C for 4 h, after that the system was allowed to cool to room temperature naturally. The precipitate was centrifuged and washed with deionized water for two times and then dried in the vacuum freeze drier at −60 °C for 24 h, before resulting in NaYF_4_:Ho^3+^@TiO_2_-rGO composites.

### Photocatalytic activity measurement

The photocatalytic activity of the NaYF_4_:Ho^3+^@TiO_2_-rGO composites was measured via comparing the concentration of rhodamine B (RhB) after irradiation to the original concentration of RhB using a Hitachi U-3010 UV-Vis spectrophotometer (Hitachi Corp., Tokyo, Japan). The percentage of degradation is indicated as C/C_0_, where C is the concentration of RhB at the irradiation time t and C_0_ is the concentration at adsorption equilibrium with the photocatalyst before irradiation. Typically, 50 mg of photocatalyst was suspended in 250 mL RhB aqueous solution (5 mg/L) by sonication. Prior to irradiation, the suspension was stirred in the dark for 0.5 h to establish the adsorption-desorption equilibrium. Then the solution was exposed to the irradiation of a500 W Xenon arc lamp with a UV cutoff filter (λ > 400 nm). Every 2 hour, 8 mL of the transparent, aqueous solution was collected and then centrifuged (10,000 r/min) prior to analysis with the Hitachi U-3010 UV-Vis spectrophotometer. As reference experiments, t the photodegradation of RhB with P25 (Degussa P25), with β-NaYF_4_:Ho^3+^, and without any catalyst were tested, respectively. All the experiments were at the same conditions for comparison.

### Characterization

The crystal structures of all prepared samples were characterized by X-ray diffraction (XRD) using a Rigaku D/Max2500pc diffractometer with Cu Kα radiation. Scanning electron microscopy (SEM) images were obtained with a Zeiss AURIGA FE microscope (EHT = 5 kV, WD = 8.8 nm; Zeiss, Oberkochen, Germany). An energy-dispersive X-ray analysis (EDS) of the samples was also performed during the SEM measurements. Transmission electron microscopy (TEM) measurements were carried out on a FEI Tecnai G20 operated at an acceleration voltage of 200 kV. The surface chemical environments were analyzed by X-ray photoelectron spectra (XPS) on a PHI5000 VersaProbe system with monochromatic Al Kα X-rays. The composite was applied with Fourier transform infrared spectroscopy analysis (FT-IR, IRPrestige-21, Shimadzu, Japan)using FT-IR spectrophotometer (KBr as the reference sample). UV-Vis diffuse-reflectance spectroscopy (UV-Vis DRS) was performed using the Hitachi U-3010 UV-Vis spectrophotometer. Raman spectra were recorded on an HR Evolution instrument with an Ar + laser source of 488 nm. The Brunauer-Emmett-Teller (BET) surface areas measurements and evaluation of porosity of the samples were conducted on the basis of nitrogen adsorption isotherms measured at 400 °C using a gas adsorption apparatus (Gemini VII 2390, Micromeritics Instrument Corp, Norcross, GA, USA). The sample for electron spin resonance (ESR) measurement was prepared by mixing NaYF_4_:Ho^3+^@TO–rGO samples in a 50 mM DMPO solution tank (aqueous dispersion for DMPO-·OH and methanol dispersion for DMPO-·O_2_^−^). Photoelectrochemical properties were evaluated using CHI Electrochemical Workstation (CHI 760E, Shanghai Chenhua, China). All the photoelectrochemical measurements were performed under Vis light of a 300 W Xe lamp coupled with 420 nmcutoff filters. Three-dimensional (3D) fluorescence spectra were obtained with a Hitachi F-7000 fluorescence spectrophotometer with a 150 W Xe lamp as excitation source. All experiments were performed at room temperature.
